# S162. IMPACT OF THE PRESENCE OF A PEER WORKER IN AN EARLY INTERVENTION UNIT FOR YOUNG ADULTS WITH MENTAL ILLNESS (JADE)

**DOI:** 10.1093/schbul/sby018.949

**Published:** 2018-04-01

**Authors:** Maryse Badan Ba, Elisabeth Sturm, Laurent Bediat, Logos Curtis

**Affiliations:** 1 University Hospital of Geneva; 2 Re-pairs Association

## Abstract

**Background:**

A current trend in health care and in particular mental health care is to reduce the divide between patients and their community, which is encouraging new practices as well as new health care professions. The concept of a peer worker, a previous mental health care user, is revealing itself to be complementary to that of other health care workers as well as effective (Davidson et al., 2012). One aspect of the peer worker given his or her previous experience is as an intermediary for communication. In mental health care units such as ours (Geneva based JADE program for early intervention in mental health) the introduction of a peer worker as a new concept can lead to many benefits but also carries questions and uncertainties.

**Methods:**

In order to assess the impact of a peer worker’s presence in our unit over a period of 2 months, we submitted questionnaires to patients and staff. We present results from questionnaires from 7 patients and 15 staff. In order to further explore the subjective appreciation of this integration, we included open ended questions to also assess constructive suggestions from patients and staff.

**Results:**

Data collection is in progress.

**Discussion:**

The impact of the presence of peer-worker in our mental health care unit will be discussed.

